# Supplementary material for: Apolipoprotein E abundance is elevated in the brains of individuals with Down syndrome–Alzheimer’s disease
Source: Acta Neuropathol. 2025 May 19;149(1):49. doi: 10.1007/s00401-025-02889-0 (PMC12089208; doi:10.1007/s00401-025-02889-0)
Supplement: Supplementary file 1 — Supplementary file1 (PDF 1650 KB) [file 401_2025_2889_MOESM1_ESM.pdf]

Apolipoprotein E abundance is elevated in the brains of individuals with Down syndrome-Alzheimer's disease

Clíona Farrell[1,2], Yazead Buhidma[2], Paige Mumford[1,2], Wendy E. Heywood[3], Jenny Hällqvist[3], Lisi Flores-Aguilar[4], Elizabeth J. Andrews[4], Negin Rahimzadah[5,6,7], Orjona Stella Taso[1,2], Eric Doran[9], Vivek Swarup[6,7,8], Elizabeth Head[4], Tammaryn Lashley[2], Kevin Mills[3], Christina E. Toomey¶[2,10], and Frances K. Wiseman¶[1,2,]

Supplementary Information

Case type and ID	Sex	Age at death (years)	Post-mortem interval (hours)
DS_1	F	1	28
DS_10	M	25	24
DS_11	M	25	22
DS_12	M	19.87	14
DS_13	M	15	14
DS_3	M	2	17
DS_4	M	23	15
DS_5	M	24	24
DS_7	F	39	12
DS_8	M	19	26
DS_9	F	3	11
YC_10	M	25	21
YC_11	M	19	14
YC_12	M	25	23
YC_13	M	23	18
YC_14	F	47.3	5
YC_15	M	2	25
YC_2	M	1.72	25
YC_3	F	2	24
YC_7	F	39	17
YC_8	M	19	24
YC_9	M	24	24

Supplementary Table 1 Case demographics for posterior cingulate cortex cases. Cases were sourced from NIH NeuroBioBank (USA). No significant difference in age at death was identified between case types (Univariate ANOVA, $F(1,20) = 0.249$, $p = 0.623$). No significant difference in PMI was identified between case types (Univariate ANOVA, $F(1,20) = 0.204$, $p = 0.656$). YC = Young control, DS = Down syndrome, M = male, F = female.

Cohort	Sample Code	Case Type	RNA Integrity Number (RIN)
Discovery	DSAD1	DSAD	6
Discovery	DSAD2	DSAD	3.9
Discovery	DSAD3	DSAD	3.8
Discovery	DSAD4	DSAD	6.9
Discovery	DSAD5	DSAD	6.3
Discovery	DSAD6	DSAD	6.5
Discovery	DSAD7	DSAD	Not available
Discovery	DSAD8	DSAD	5.8
Validation	DSAD9	DSAD	3
Validation	DSAD10	DSAD	5.9
Validation	DSAD11	DSAD	5.1
Validation	DSAD12	DSAD	3.7
Validation	DSAD13	DSAD	6.1
Validation	DSAD14	DSAD	5.9
Validation	DSAD15	DSAD	3.4
Validation	DSAD16	DSAD	4.3
Validation	DSAD17	DSAD	6.8
Validation	DSAD18	DSAD	6.7
Discovery	HA1	HA	Not available
Discovery	HA2	HA	7.4
Discovery	HA3	HA	6
Discovery	HA4	HA	3.9
Validation	HA5	HA	3.2
Validation	HA6	HA	2.9
Validation	HA7	HA	5
Validation	HA8	HA	6
Validation	HA9	HA	5.8
Validation	HA10	HA	4.3
Validation	HA11	HA	6.2
Validation	HA12	HA	5.9
Validation	HA13	HA	2.6
Validation	HA14	HA	6.9
Discovery	EOAD1	EOAD	4.6
Discovery	EOAD2	EOAD	3.5
Discovery	EOAD3	EOAD	2.3
Discovery	EOAD4	EOAD	4.8
Validation	EOAD5	EOAD	2.3
Validation	EOAD6	EOAD	3.5
Validation	EOAD7	EOAD	3.5
Validation	EOAD8	EOAD	3.1
Validation	EOAD9	EOAD	1.9
Validation	EOAD10	EOAD	2
Validation	EOAD11	EOAD	3.3

Validation	EOAD12	EOAD	3.3
Validation	EOAD13	EOAD	4.3
Validation	EOAD14	EOAD	5.3

Supplementary Table 2 RNA Integrity Numbers are variable in human brain samples. RNA Integrity Numbers (RIN) were measured using an Agilent Tapestation, with a score of 0 indicating completely degraded RNA, and 10 being fully intact RNA. Across both discovery and validation samples, RIN was variable, and was affected by age, sex or PMI, but an effect of case type (Univariate ANOVA, $F(2,35) = 8.967$, $P < 0.001$) and Braak stage (Univariate ANOVA, $F(1,35) = 5.453$, $P = 0.025$) were found on RIN. EOAD had the lowest mean RIN at 3.4, with AD-DS and CTRL samples having mean RIN of 5.1 and 5.3 respectively.

Experiment (Case types)	Cohorts and Source	Figures
Bulk-proteomics (DSAD, HA, EOAD)	Discovery (NBTR)	Figure 1, Tables 4-7
Single-nuclei RNA-sequencing (DSAD, HA, EOAD)	Discovery (NBTR)	Figure 2
Difference in APOE abundance (DSAD, HA, EOAD)	Discovery (NBTR)<br>Validation A (SWDBB)	Figure 3 a, b, e, f, I, j, k
Difference in APOE abundance (DSAD, HA LOAD)	Validation B (ADRC-UCI and NIH)	Figure 3 c, d, g, h, l, m
Relationship between APOE and *APP* gene products (DSAD, HA, EOAD)	Discovery (NBTR)<br>Validation A (SWDBB)	Figure 4, supplementary table 4
Relationship between APOE and Tau (DSAD, HA, EOAD)	Discovery (NBTR)<br>Validation A (SWDBB)	Figure 5

Supplementary Table 3 Summary of cohorts used for each experiment. Summary table of the principal experiments, the cases used, the source of these samples, and the figures and tables reporting the results.

**Fraction and *APP* product**		**p**	**R**
Total	APP-CTF-α	0.0026	0.4473
	APP-CTF-β	0.0247	0.3422
	FL-APP	0.0001	0.6193
5M Gnd HCl	Amyloid-β_{42}	0.2878	0.1701
	Amyloid-β_{40}	0.0041	0.4802
1% Triton	Amyloid-β_{42}	0.0047	0.4227
	Amyloid-β_{40}	0.0015	0.4696
TBS	Amyloid-β_{42}	0.0122	0.3791
	Amyloid-β_{40}	0.0053	0.4178

p	Colour
<0.0001	
<0.001	
<0.01	
<0.05	
<.1	
> .1	

Supplementary Table 4 APOE abundance (Sigma) by western blot correlates with APP and its processing products in discovery and validation cohort A. As in Figure 4, correlation analysis was carried out between APOE abundance (Sigma, SAB2701946) by western blot and APP / APP-CTFs (Y188, Abcam) by western blot, and amyloid-β40 / amyloid-β42 by MSD assay. P-value and Pearson's R shown. Significant positive correlations are shown in red, with increased intensity of colour representing a more significant relationship. Discovery and validation cohort A, n=14 HA, n=18 DSAD, n=14 EOAD.

Supplementary Figures

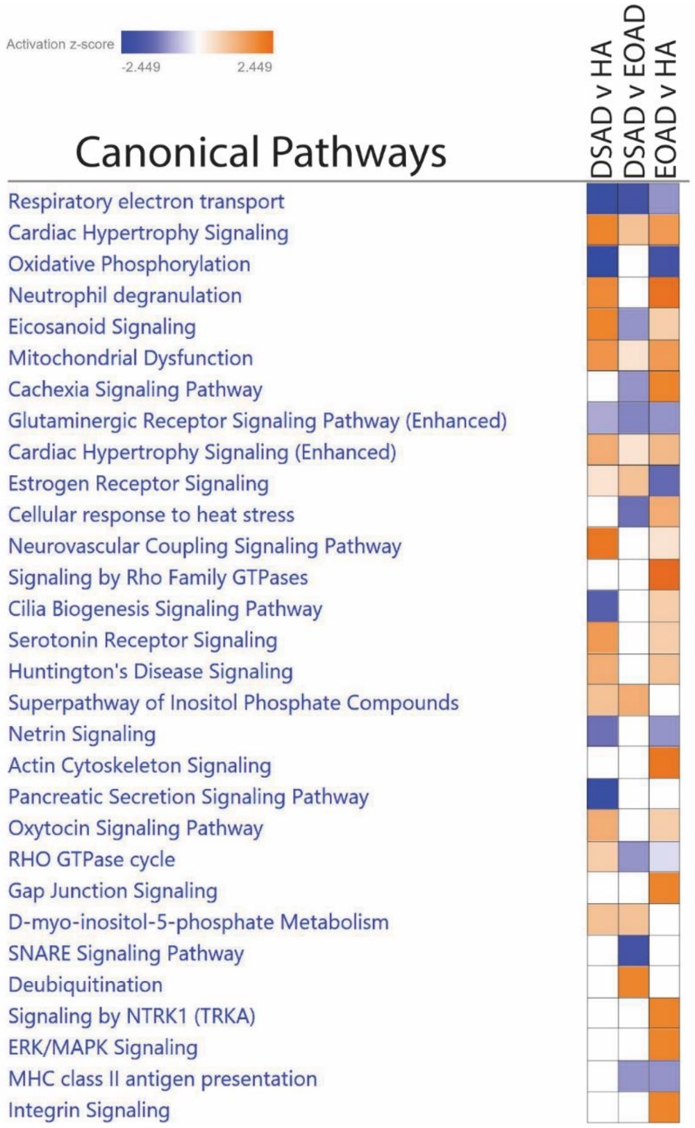

Supplementary Figure 1 Canonical pathway analysis of the proteomics dataset. IPA was used to assess the altered canonical pathways between DSAD v HA, DSAD v EOAD and EOAD v HA. The top 30 enriched pathways are shown for each comparison. In IPA, a z-score was assigned to each pathway based on the predicted direction of change of the pathway based on the number of proteins from our dataset overlapping with the pathway. A positive z-score indicates the pathway is likely to be increased in activity (orange), and a negative z-score indicates the pathway is likely to be decreased in activity (blue). The colour intensity of the bar indicates the confidence in the predicted direction. White boxes have a neutral z-score meaning proteins in the pathway are over-represented in the dataset, but do not have a uniform directional change in abundance. Discovery cohort, n=4 HA, n=8 DSAD, n=3 EOAD.

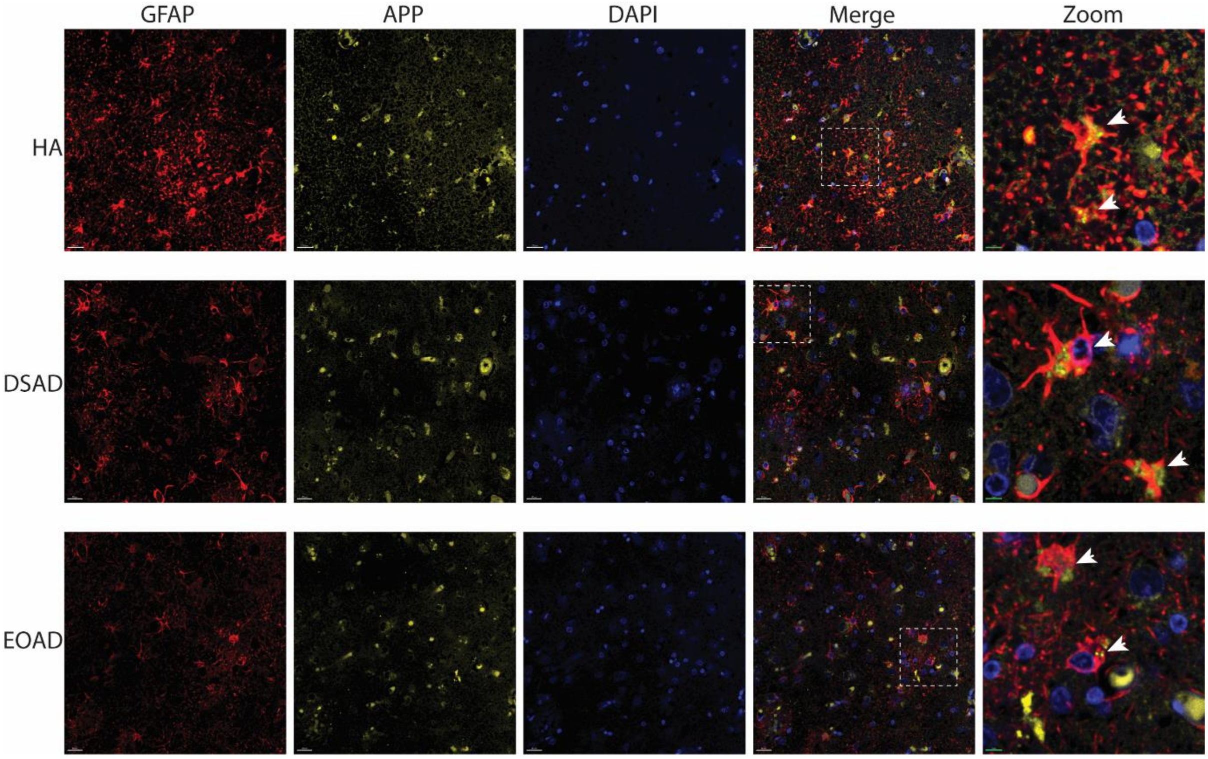

Supplementary Figure 2 APP can be expressed in GFAP positive cells in HA, DSAD and EOAD cases. Representative immunofluorescence co-staining of APP and astrocyte marker GFAP in post-mortem tissue sections from HA, DSAD and EOAD cases. GFAP (red), APP (yellow) and DAPI (blue). Across all cases, co-labelling of the same cell with APP and GFAP is observed (white arrowheads in zoom panel – area denoted by dashed white line). Cases in representative images are HA2, DSAD5 and EOAD3. White scale bars = 20 μm. Green scale bars on zoom images = 5 μm.

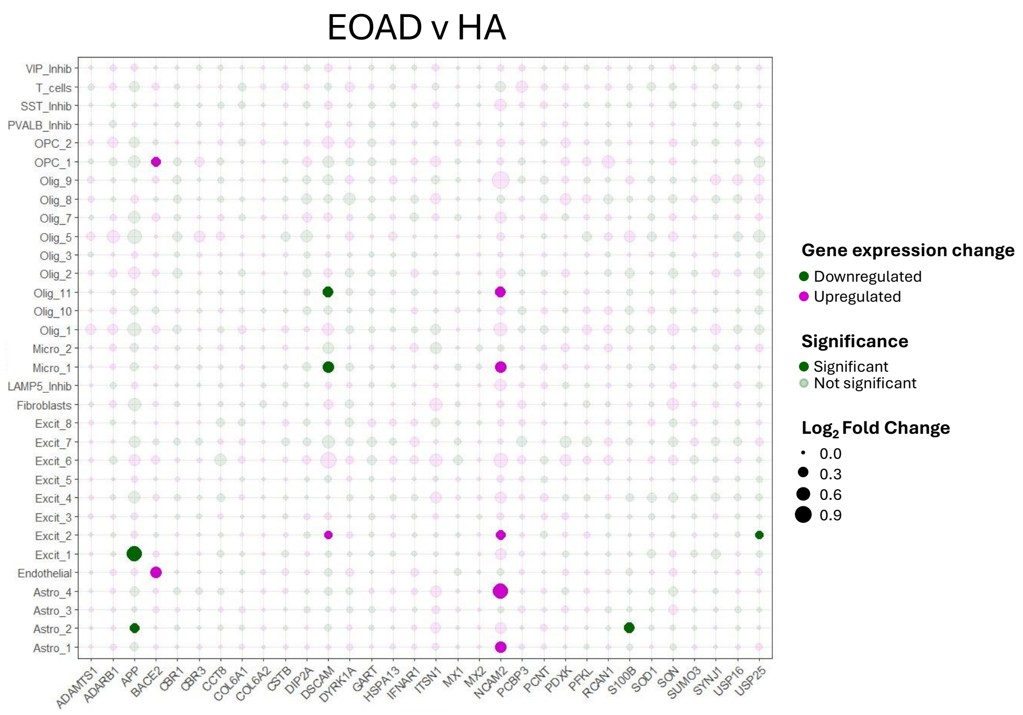

Supplementary Figure 3 Differential gene expression analysis of Hsa21 genes in EOAD vs healthy ageing cohort (HA). Hsa21-encoded genes identified in the proteomics dataset, and other commonly investigated Hsa21 genes, are dysregulated in few cell types in EOAD compared to HA. Discovery cohort n=4 HA, n=8 DSAD, n=4 EOAD.

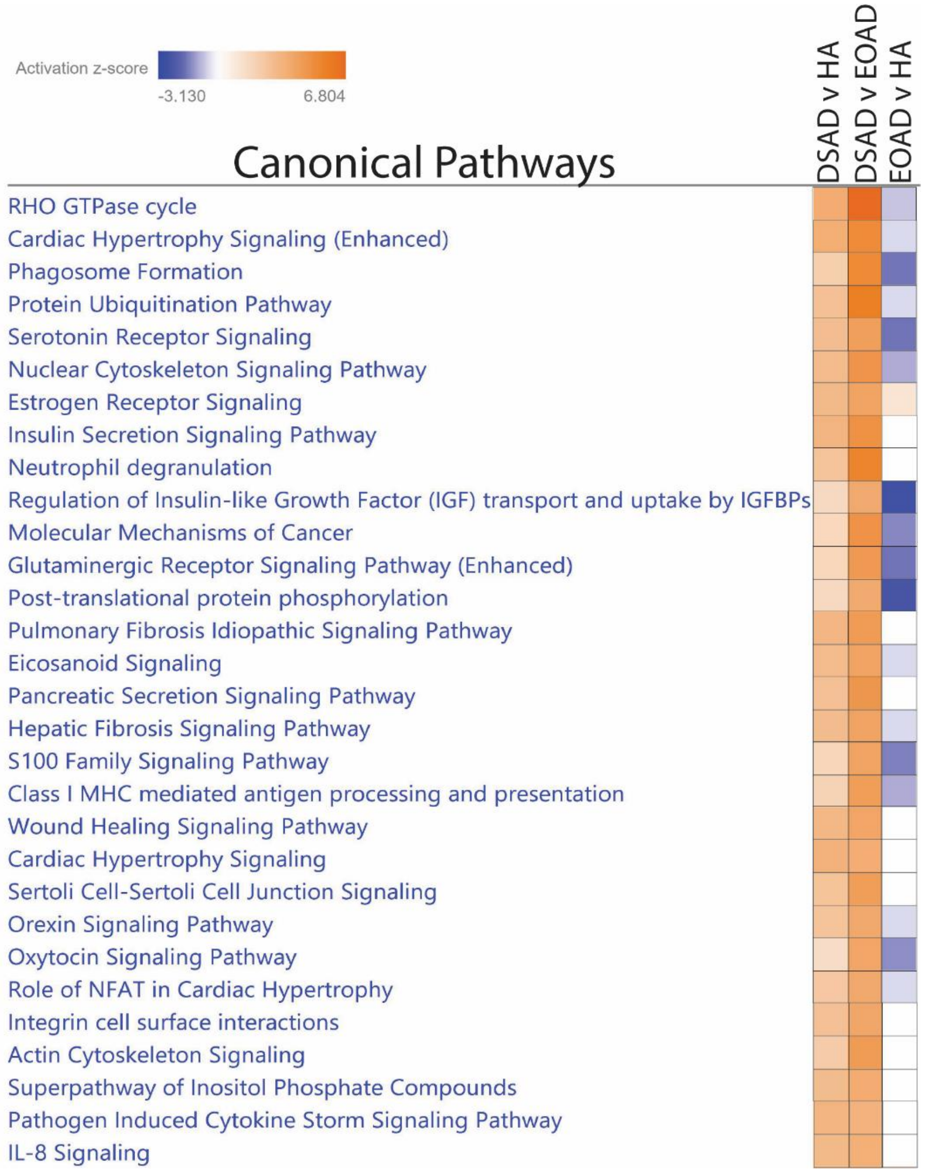

Supplementary Figure 4 Comparison heatmap of canonical pathway analysis of astrocyte 2 cluster. IPA was used to assess the altered canonical pathways between DSAD v HA, DSAD v EOAD and EOAD v HA in astrocyte cluster 2 of the snRNAeq dataset. The top 30 enriched pathways are shown. In IPA, a z-score was assigned to each pathway based on the predicted direction of change of the pathway based on the number of transcripts from our dataset overlapping with the pathway. A positive z-score indicates the pathway is likely to be increased in activity (orange), and a negative z-score indicates the pathway is likely to be decreased in activity (blue). The colour intensity of the box indicates the confidence in the predicted direction. White boxes imply a neutral z-score meaning transcripts in the pathway are over-represented in the dataset, but do not have a uniform directional change in abundance. Discovery cohort, n=4 HA, n=8 DSAD, n=4 EOAD.

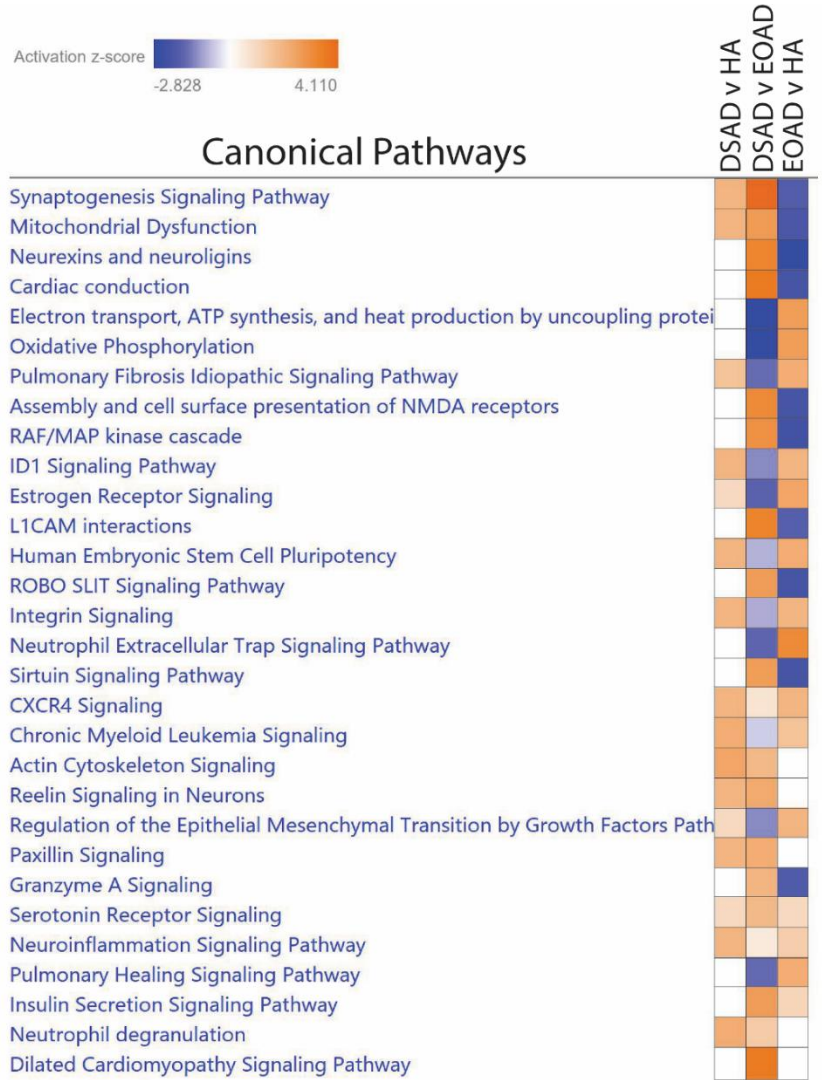

Supplementary Figure 5 Comparison heatmap of canonical pathway analysis of endothelial cells. IPA was used to assess the altered canonical pathways between DSAD v HA, DSAD v EOAD and EOAD v HA in the endothelial cell cluster of the snRNAeq dataset. The top 30 enriched pathways are shown. In IPA, a z-score was assigned to each pathway based on the predicted direction of change of the pathway based on the number of transcripts from our dataset overlapping with the pathway. A positive z-score indicates the pathway is likely to be increased in activity (orange), and a negative z-score indicates the pathway is likely to be decreased in activity (blue). The colour intensity of the box indicates the confidence in the predicted direction. White boxes imply a neutral z-score meaning transcripts in the pathway are over-represented in the dataset, but do not have a uniform directional change in abundance. Discovery cohort, n=4 HA, n=8 DSAD, n=4 EOAD.

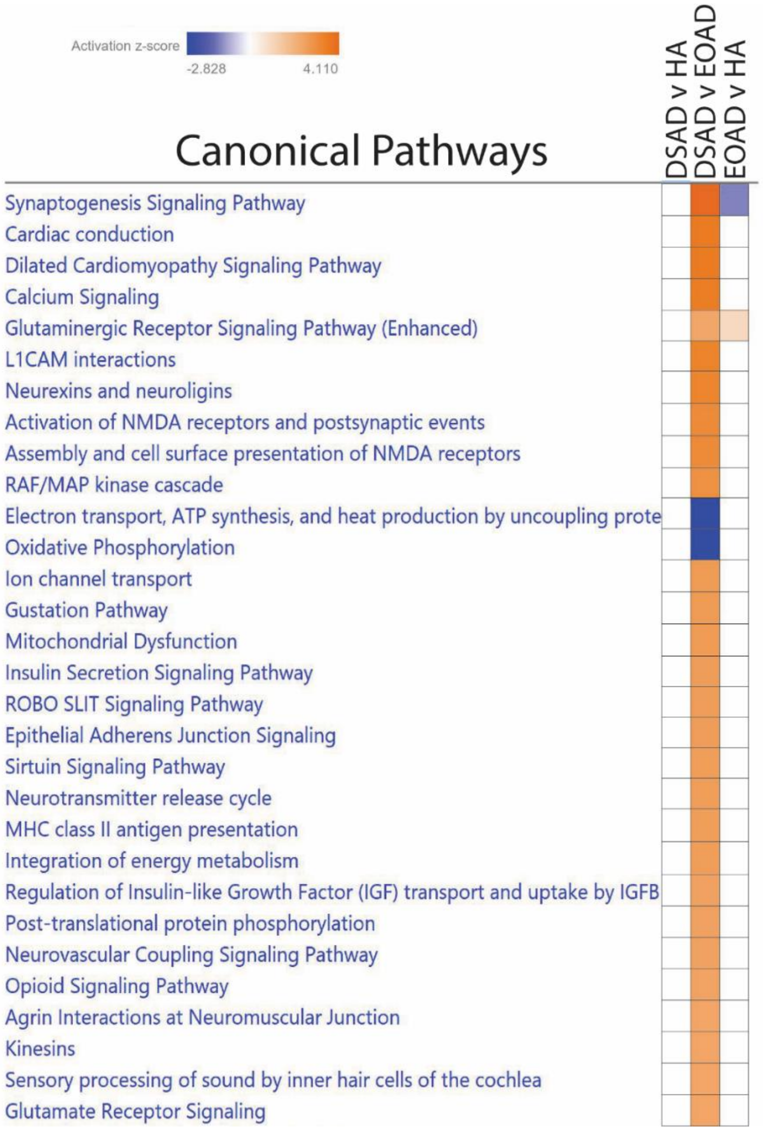

Supplementary Figure 6 Comparison heatmap of canonical pathway analysis of pericyte cell cluster. IPA was used to assess the altered canonical pathways between DSAD v HA, DSAD v EOAD and EOAD v HA in the pericyte cluster of the snRNAeq dataset. The top 30 enriched pathways are shown. In IPA, a z-score was assigned to each pathway based on the predicted direction of change of the pathway based on the number of transcripts from our dataset overlapping with the pathway. A positive z-score indicates the pathway is likely to be increased in activity (orange), and a negative z-score indicates the pathway is likely to be decreased in activity (blue). The colour intensity of the box indicates the confidence in the predicted direction. White boxes imply a neutral z-score meaning transcripts in the pathway are over-represented in the dataset, but do not have a uniform directional change in abundance. Discovery cohort, n=4 HA, n=8 DSAD, n=4 EOAD.

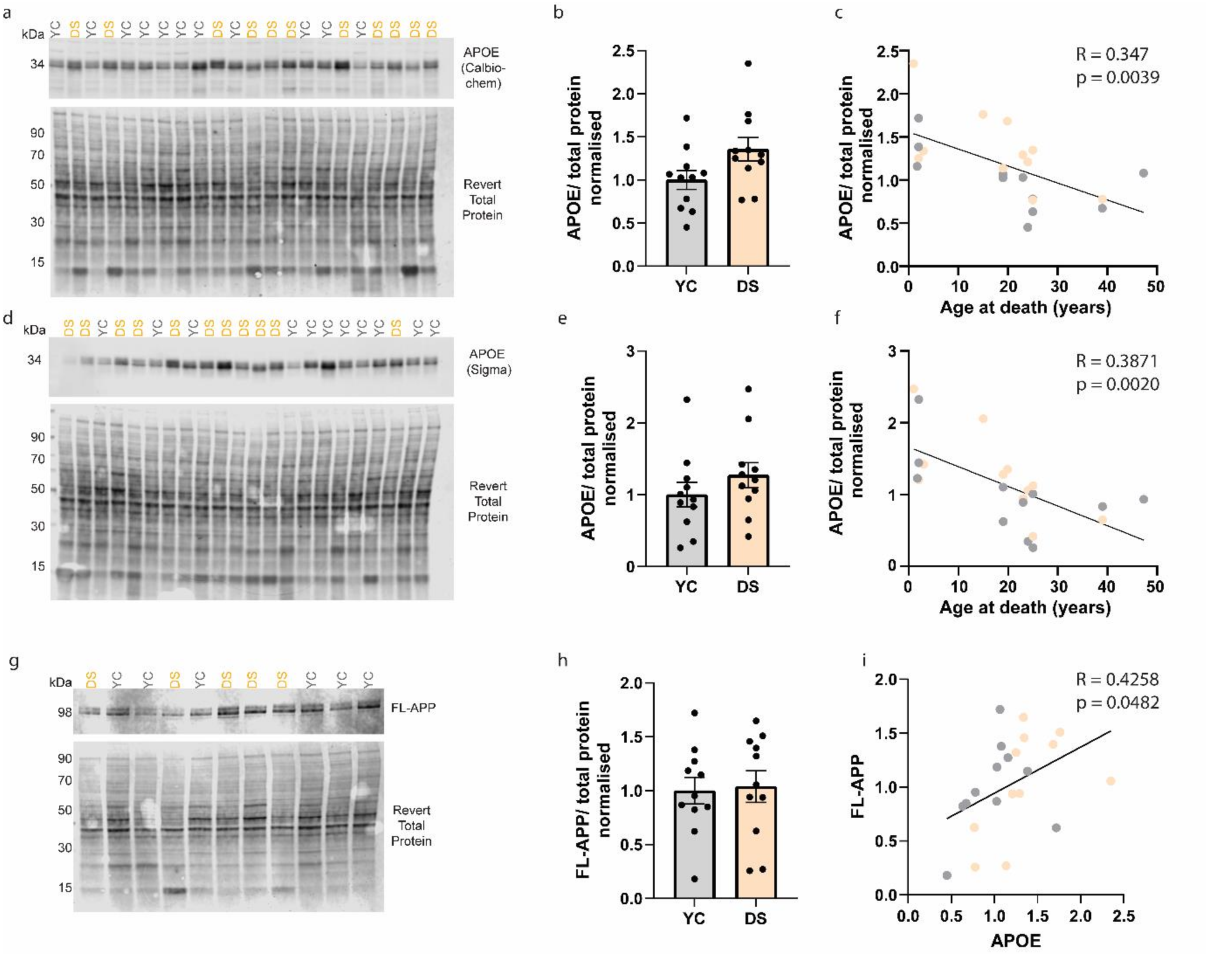

Supplementary Figure 7 APOE is not different between young control and young DS posterior cingulate cortex samples.

(a, d) Representative western blots for APOE (Calbiochem, 178479), APOE C-terminal (Sigma, SAB2701946) and Revert 700 total protein stain (Licor bio, 926-11016) in posterior cingulate cortex samples from young control (YC) and young DS (DS) (n=11 per group). (b) Case type has no significant effect on APOE abundance (Calbiochem) (Univariate ANOVA, $F(1,16) = 1.522$, $p = 0.235$), age at death significantly affected APOE abundance (Univariate ANOVA $F(1,16) = 12.961$, $p = 0.002$), no effect of sex or PMI was observed. (c) APOE (Calbiochem) and age at death (years) negatively correlate in young posterior cingulate cortex samples (Slope = -0.0972 Pearson's R = 0.3473, $F(1,20) = 10.64$, $p = 0.0039$). (e) Case type has no significant effect on APOE abundance (Sigma) (Univariate ANOVA, $F(1,16) = 0.014$, $p = 0.909$), age at death (Univariate ANOVA $F(1,16) = 17.845$, $p = 0.001$) and sex (Univariate ANOVA, $F(1,16) = 8.005$, $p = 0.012$) significantly affected APOE abundance, no effect of PMI was observed.(f) APOE (Sigma) and age at death (years) negatively correlate in young posterior cingulate cortex samples (Slope = -0.02739 Pearson's R = 0.3871, F(1,20)

= 12.63, p = 0.0020). (g) Representative western blot for APP (Abcam, Y188) and Revert 700 total protein stain (Licor Bio, 926-11016) in posterior cingulate cortex samples. (h) Case type has no effect on FL-APP abundance (Univariate ANOVA, F(1,16) = 1.89, p = 0.670), PMI significantly affected FL-APP abundance (Univariate ANOVA, F(1,16) = 5.298, p = 0.035), no effect of age at death or sex was observed. (i) APOE (Calbiochem) and FL-APP abundances positively correlate in young posterior cingulate cortex samples (Slope = 0.4278, Pearson’s R = 0.4258, F(1,20) = 4.430, p = 0.0482). Posterior cingulate cortex cases n=11 YC, n=11 DS. Data expressed as mean ± SEM.

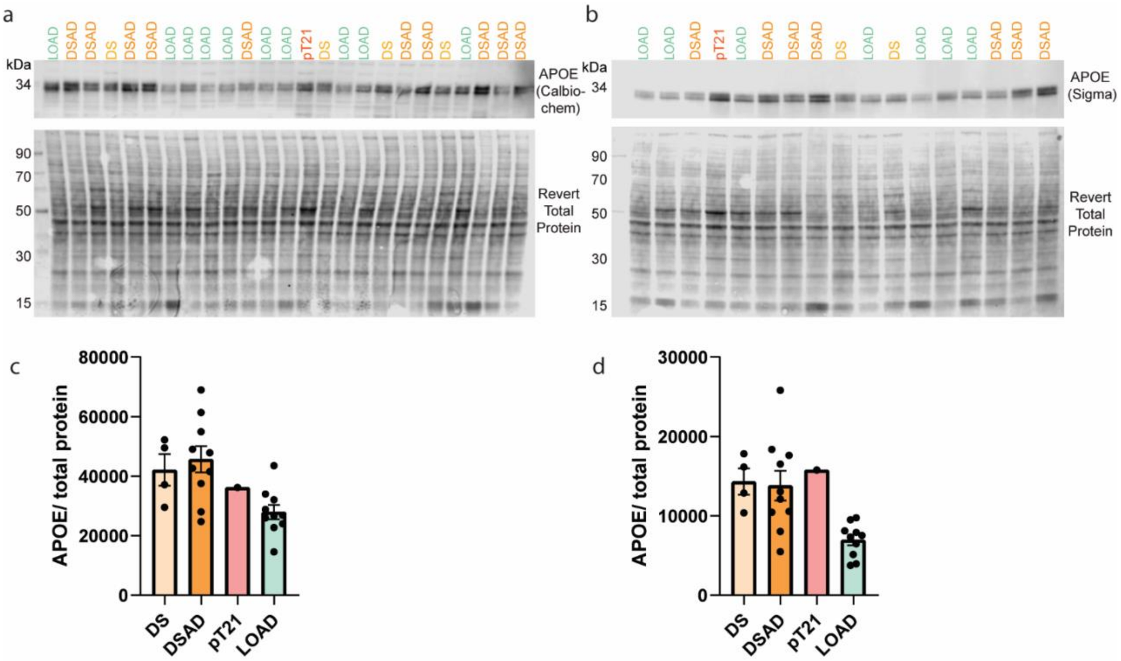

Supplementary Figure 8 APOE abundance in frontal cortex of partial trisomy 21 case. (a,b) Representative western blots for APOE (Calbiochem, 178479), APOE C-terminal (Sigma, SAB2701946) and Revert 700 total protein stain (Licor bio, 926-11016) in frontal cortex samples (validation cohort B) (n=4 DS, n=10 DSAD, n=1 pT21, n=10 LOAD). (c, d) As no young controls were included in this experiment, data is normalised to total protein only, and not internally normalised to young control values. Graphs for representative purposes only. (c, d) Validation cohort B n=4 DS, n=10 DSAD, n=1 pT21, n=10 LOAD. Data expressed as mean ± SEM.

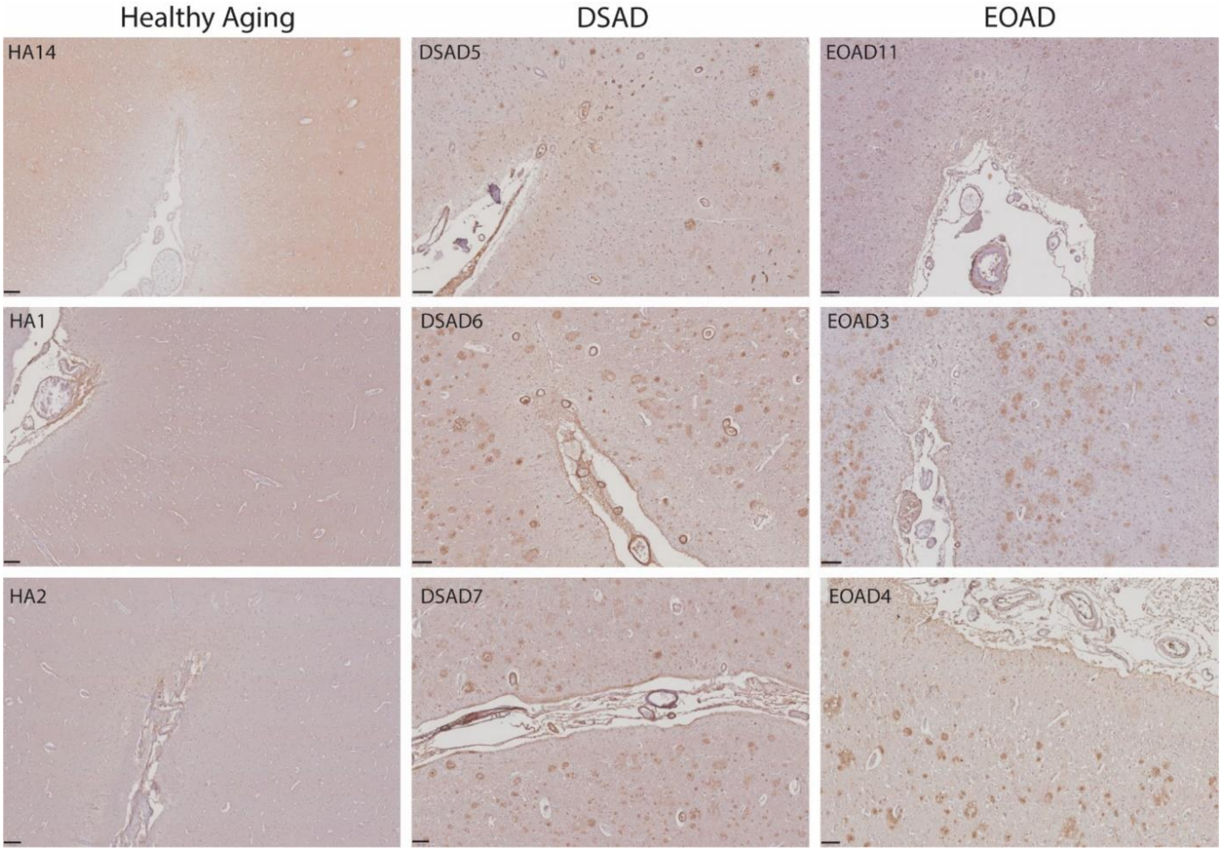

Supplementary Figure 9 APOE stains AD pathology and blood vessels in DSAD cases. Representative images of immunohistochemistry for APOE in three cases of healthy ageing (HA), DSAD and EOAD. Minimal staining is observed in HA cases. In DSAD cases, APOE staining is observed in vascular structures and structures resembling amyloid-β plaques pathology. In EOAD cases, APOE staining is primarily observed in structures resembling amyloid-β plaques pathology. Scale bar = 100 μm.
